# Institutional Housekeeping

**Published:** 1912-03-02

**Authors:** 


### INSTITUTIONAL HOUSEKEEPING.

**(Workers' Questions and Criticism of every kind will be welcomed.)**

### The Purchase and Testing of Cocoa.

Cocoa, which closely resembles tea and coffee in the peculiarity of its active alkaloids, has a much milder action upon the respiratory and nervous system than either. This probably accounts for so much less of it being consumed. Cocoa contains considerable nutriment, being more of a food than a beverage; in fact, in the seventeenth century a religious controversy took place as to whether or not cocoa were food or drink, and hence whether it was permissible to use it during fasts. Happily for the fasters the decision was given in favour of its consumption. Cocoa is imported into this country from many sources, but that from Venezuela—the Caracas cocoa—is usually considered the best. So few persons buy cocoa in the raw state that it would be of little value to dwell upon the varieties and values of the cocoa bean, which has to undergo many manufacturing processes before it is fit for sale and use as food. It is then offered in several different forms as cocoa-nibs (the purest form), flake cocoa, rock cocoa, cocoa essence, soluble cocoas. The two latter are most largely sold, for though the first-named is actually the purest, being simply the whole kernel, but without its husk, the nibs contain so large a proportion of cocoa-butter that the beverage is too rich for delicate digestions and has sometimes a coarse, even rank flavour. Another disadvantage is the length of time needed for boiling the nibs, when cocoa is required as a beverage. Flake cocoa is prepared by grinding the whole roasted bean and reducing it to a rough paste which is then dried. It is usually sold in three grades, the last two containing an excessive amount of ground husk. Rock cocoa is prepared in a very similar manner, but some of the cocoa-butter is removed and a soft smooth paste is made by the admixture of sugar and starch. The other forms consist mainly of the carefully prepared and powdered beans from which some of the heavy cocoa-butter has been previously expressed. Sometimes starch, usually arrowroot and sugar are added, and in some of the cheaper varieties this is done to excess. If these mixtures are used the beverage should be boiled for a minute or two in order to cook the starch granules. To speak of these cocoas as soluble is not strictly speaking correct, as the cocoa does not dissolve but remains suspended in the thick mucilage. Many Dutch cocoas are treated with some form of alkali to render the albuminoids more soluble and soften the fibre. This practice is however not advocated by manufacturers in this country, though many question if it is in any way harmful. The actual signs by which good and inferior cocoas can be judged are almost nil, the wisest plan being to avoid very low-priced varieties which probably contain adulterants in the form of ground cocoa-husks. Out of 695 samples of cocoa examined by the inspectors of the Local Government Board in 1910-11, 14 per cent. were reported against, principally on account of containing large quantities of powdered cocoa-shell. The presence of an undue quantity of added starch may be detected by noting if the beverage thickens unduly when boiled. Should there be reason to suspect treatment with an alkali it may be detected by putting a slip of red litmus paper into a solution of the powder. Cocoa requires to be stored in a perfectly dry, fairly cool place in air-tight tins or bottles, and not in the proximity of any strongly flavoured foods.

